# Environmental impacts of industrial activities on floral coverage with special emphasis on detoxification enzyme activities in *Cataglyphis savignyi* as pollution biomarker

**DOI:** 10.1007/s11356-023-30367-1

**Published:** 2023-10-18

**Authors:** Yasser I. Hamza, Ahmed S. Bream, Mohammed A. Mahmoud, Mohamed A. M. El-Tabakh

**Affiliations:** Zoology Department, Faculty of Science, Al-Azhar University, Cairo, Egypt

**Keywords:** Industrial activities, Floral coverage, Detoxification enzyme activities, *Cataglyphis savignyi*

## Abstract

The present study investigates the environmental impact of industrial activities on floral coverage within the major industrial district of Borg El-Arab City, Egypt. Additionally, it aims to evaluate the detoxification enzymatic activity of *Cataglyphis savignyi* as a pollution biomarker. To achieve this objective, seasonal soil samples were collected from the studied sites to determine soil properties and heavy metal concentrations. Furthermore, a seasonal specimen of *C. savignyi* was collected to study the enzymatic activity of acetylcholinesterase (AChE) and glutathione-S-transferase (GST). Heavy metal contamination pollution indices were calculated, and fourteen plant species were identified at the investigated sites for four successive seasons from 2020 to 2021. The soil physicochemical parameters significantly varied in the industrial sites compared to the control site. The accumulation of heavy metal contamination in soil for investigated sites followed the order Ni > As > Pb > Hg. Calculated Cdeg and PLI for industrial 3 revealed a very high degree of contamination, attributed to increased industrial activity from the chemical and silicate factories that characterize this region. The current results highlight the inhibition of GST levels in *C. savignyi* at the industrial site compared to the control site. In contrast, AChE increases, which might be due to heavy metals enhancing acetylcholine activity at synapses. Consequently, the antioxidant enzymatic activities are useful as biomarkers for assessing and monitoring environmental contamination. In conclusion, this study underscores insects as potent biomarkers for heavy metal contamination, marking a significant advancement in environmental monitoring. These bioindicators offer crucial insights into the impacts of climate change and industrial pollution. The research reveals distinct plant diversity variations and higher heavy metal content in industrial sites, indicating pronounced contamination. Additionally, the study highlights altered enzyme activities in insects, emphasizing their utility as biomarkers for assessing environmental contamination. This work represents a substantial leap forward in comprehending the complex dynamics between contamination and ecological balance.

**Supplementary Information:**

The online version contains supplementary material available at 10.1007/s11356-023-30367-1.

## Introduction

Climatic changes, forest clearing, intensive agriculture, global trade, and pollution are the primary synthetic defendants of Earth’s life transformations. Recently, evaluating the status of the surrounding environment and its ecosystem has been a growing and necessary demand for all scientists, especially with global changes. The industrial revolution, intensification, and technological advancement since the eighteenth century (Maja & Ayano 2021) were major energy sources for humans’ impact on natural ecosystems. The greatest concerns of this development are the emissions of heavy metals and other harmful substances, which have permanent and unpredictable worldwide environmental impacts. As a result, they have a detrimental influence on animal populations and seem disruptive to ecosystems (Perdigão & da Silva Pereira 2021). Heavy metals in soils may be beneficial to the ecology or detrimental, depending on their concentration. Many common effects of various metals on diverse plants might occur from metal overload. Several of these elements could be necessary for the biota in insignificant amounts, but there might be toxicity issues at greater concentrations.

Heavy metal poisoning of soils has become a major problem for governments and regulatory agencies concerned with environmental and human risk assessment (Chonokhuu et al. 2019). The importance of this type of contamination arises from the toxicity and persistence of heavy metals, long-range transport ability via food webs or food chain, and bioaccumulation in human and animal tissue, which is associated with the long-term viability of ecological systems (Emenike et al. 2021). As a result, soil may be seen as a bed reservoir for contaminants, a heavy metal accumulator, and an excellent indicator of environmental quality (Danesino 2009).

Some authors described the transmission of heavy metals to soils (Zeng et al. 2011; Mandal and Voutchkov 2011; Soriano et al. 2012), which are taken up as aerosols by the atmosphere to several kilometers distant from their sources and transmitted to the soil via wet or dry deposition. As a result, heavy metals in soils have been identified as strong tracers for monitoring the effect of human activities, such as industrial emissions (cement plants, fossil fuel, and coal combustion chemical plants), automotive emissions, and atmospheric deposition. These result in heavy metal emissions into the atmosphere and subsequent soil deposition (Guo et al. 2012; Lu et al. 2012). These potentially dangerous chemicals come into direct touch with clays and organic material in soil, both of which have a high capacity for binding to chemical compounds and substances (Yang et al. 2021) and changes in the main properties of soil.

Soil management may also affect its physical, chemical, and biological properties and detect varied reactions by biological activities such as enzymatic activity to heavy metal toxicity. Furthermore, high metal concentrations may interfere with the operations of bacteria that contribute to plant growth (Singh et al. 2020). In high quantities, all heavy metals are harmful and are considered environmental pollutants (Yaashikaa et al. 2022). Heavy metals are potentially hazardous to plants, causing chlorosis, sluggish plant development, yield depression, and maybe decreased nutrient absorption, plant metabolic abnormalities, and, in leguminous plants, a reduced capacity to fix molecular nitrogen. Heavy metal soil pollution reduces agricultural productivity and has serious health consequences since it enters the food chain (Singh & Singh 2020).

Anthropogenic pollution often significantly impacts phytocoenoses because plants cannot resist the impacts of stress and must change their physiological, biochemical, anatomical, and morphological systems to cope. In addition to being very sensitive to environmental disturbances, vegetation also visibly reflects these disturbances caused by anthropogenic activity by changing several properties. Analysis of heavy metal accumulations is crucial for researching how heavy metals affect plants (Popova 2016). Because of their effectiveness in the destruction of various xenobiotics and their antioxidant activity in the protection of essential molecules such as DNA, RNA, and proteins, enzymes were an early sensitive and responsive instrument extensively utilized in the evaluation of environmental pollution (Domingues et al. 2010; Praetet al. 2014). GSTs, a detoxifying enzyme superfamily, play an important role in detoxification by catalyzing the conjugation of reduced glutathione (GSH) with electrophilic endogenous and xenobiotic compounds such as chemosynthetic insecticides and natural phytochemicals, converting them to less toxic water-soluble forms (Grant and Matsumura 1989; Singh et al. 2001). Acetylcholinesterase (AChE) is a nerve impulse transmission enzyme, and its suppression is a well-established indicator of neurotoxicity induced by organophosphate and carbamate pesticide exposure (Galgani and Bocquene 1991; Jebali et al. 2006); however, new research indicates that it may also reflect overall stress (Lavado et al. 2006). These biomarkers were selected to indicate the environmental bioavailability of the pollutants.

The research’s essential aim was to monitor the habitat characteristics through climatic parameters, soil quality (physico-chemical parameters), flora, and fauna in Borg El-Arab City, Egypt. The activity of detoxifying enzymes in *Cataglyphis savignyi* was detected as a biochemical indicator and a species’ response tool to pollution.

## Materials and methods

### Study area

The Borg El-Arab district is geographically bounded north by the Mediterranean Sea, south by tableland, east by El-Amerya township, and west by El-Hammam small town (Kassas 1972).

**Fig. 1:**
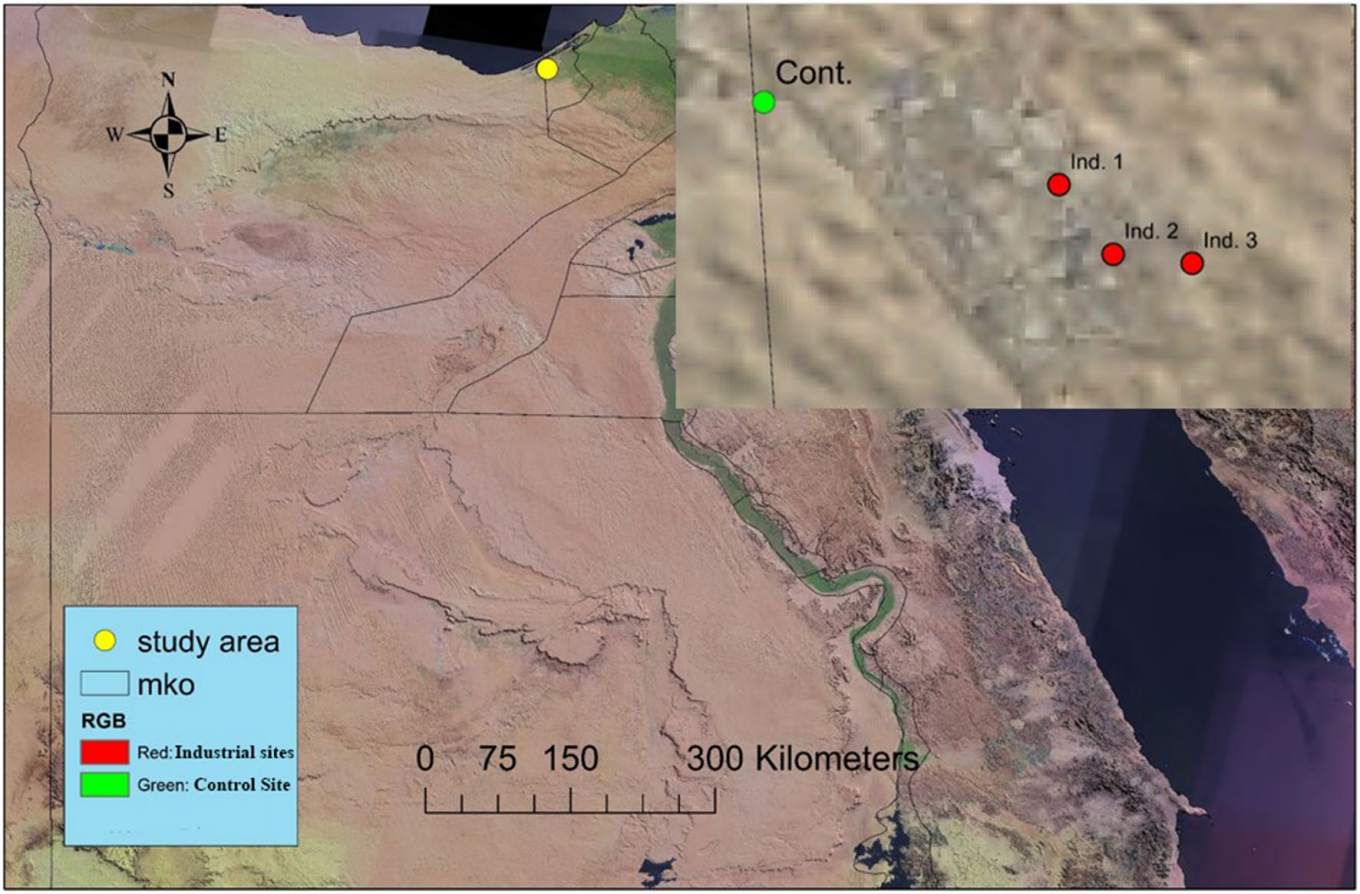
Map of investigated sites

Seasonal sampling of ground fauna was carried out at three industrial sites and one control site (located at least 4 km from the nearest anthropogenic activity) for four successive seasons during 2021. The coordinates of each site (Fig. 1) were recorded using a hand-held Global Positioning System (Garmin, GPS III plus) and a description of soil and plant habitat parameters.

- Cont.: lies at N 30° 52′ 58.2″–E 29° 35′ 40.2″ at the position of New Borg El-Arab Bus Stop to Cairo and Alexandria, which is characterized by no human impact and natural habitat in a desert region.
- Ind. (1): lies at N 30° 52′ 37.4″–E 29° 36′ 54.3″ at 1st Industrial Zone, Bahig, Borg El-Arab Al Gadida City, Alexandria Governorate. This site represents the community of different types of factories such as El-Amriafor Rubber, Plastic Industry (Faroplast), Center for Medical Supplies, EL-Tawheed for advanced industries (Sparkle shampoo and conditioner), and Fredex Firefighting & Metal Products.
- Ind. (2): lies at N 30° 52′ 19.9″–E 29° 37′ 07.9″ at 2nd Industrial Zone, Bahig, Borg El-Arab Al Gadida City, Alexandria Governorate. This site represents the community of different types of factories, such as Macris Silicates Factory, Elola Steel Group, and Arabian Mills Company.
- Ind. (3): Lies at N 30° 52′ 17.6″–E 29° 37′ 27.7″ at 2nd Industrial Zone, Bahig, Borg El-Arab Al Gadida City, Alexandria Governorate. This site represents the community of different types of factories such as Targo Chem for chemical industries, Borg El-Arab Industries–Abaza, National Co. for Oil, Sika-Borg El-Arab Factory, GreasePentra paints Company, ACC Construction Chemicals, and Al Asdeqa Dairy Factory.

### Climatic factors

Climatic data were obtained from the meteorological station in Borg El-Arab. The seasonal mean of average air temperature degree (°C), pressure (kPa), wind speed (m s^−1^), and Rh (%) were the main climatic parameters.

### Soil sampling and analysis

Soil samples were obtained independently from several research locations, according to Carter and Gregorich (2008).

#### Physical analysis

Mechanical analysis was used on soil samples to evaluate particle sizes and, lastly, the texture of the soil samples. The relative percentage between sieving sizes was used to express the soil type classes (Piper 1947; Jackson 1962a, b).

#### Chemical properties

Clear soil extract was obtained according to (Jackson 1967). Organic matter %, CaCO_3_%, pH-values, electrical conductivity (EC), soluble anions CO_3_^−−^, HCO_3_^−^, Cl_2_^−^, SO_4_^−−^, and soluble cations Ca^++^, Mg^++^, Na^+^, and K^+^ were determined in the 1:5 soil water extract as follows.

Titration against 1.0 N HCl was used to determine calcium carbonate, as stated by Allen et al. (1976). The amount of oxidizable organic carbon (as an indicator of total organic matter) was calculated using Walkley and Black (1934) rapid titration method as described by Black (1965). A PH meter was used to measure the concentration of hydrogen ions in the soil solution (Brower and Zar 1984). The total dissolved salts of soil samples were measured by a T.D.S meter (Rowell 1994). An electrical conductivity meter measured the soil’s electrical conductivity (E C) (Rowell 1994).

#### Determination of soluble anions

Carbonate ions were determined in the 1:5 soil solution extract by titration against 0.1N HCl and phenolphthalein as an indicator (Rowell 1994). On the other hand, bicarbonate ions were determined in the 1:5 soil solution extract by titration against 0.1 N HCl and methyl orange as an indicator (Rowell 1994).

#### Sulfates

According to Jackson (1967), the sulfate content was estimated gravimetrically.

#### Chlorides

Chloride ions were determined by titrating as described by Brower and Zar (1984).

#### Determination of soluble-cations

Cations of sodium and potassium were determined in the soil extract by using Varian vista AX CCD Simultaneous ICP-AES and expressed as ppm dry weight (EPA 2007). The extractable cations of Ca^++^ and Mg^++^ were determined by titration method according to Richards (1954); values were expressed as meq/L.

#### Floral coverage

Identifying the plant species associated with the different types of study sites. Therefore, plant species were collected, preserved, and pressed until identification. The nomenclature of the plant species was identified according to key authors (Tackholm 1947; Boulos 1999 & 2000 & 2002 & 2005 & 2008). According to Shukla and Chandel (1989), the relative value of vegetation cover for each species was calculated by counting the cover of each species to the total vegetation cover within a series of randomly distributed stands.

#### Heavy metal analysis

For estimation of the heavy metals (As, Pb, Ni, and Hg) in soil, the soil was digested based on the protocol of Perchloric Acid–Nitric Acid Digestion described by (Hesse 1971) and analyzed by Varian vista AX CCD Simultaneous ICP-AES and expressed as ppm dry weight.

#### Pollution indices

Different pollution indices were used for the detection of the status of pollution.

#### Contamination factor

The contamination factor and degree were also used to evaluate soil pollution. The version proposed by Hakanson (1980) allows for evaluating soil pollution by comparing concentrations in the soil’s surface layer to preindustrial levels.

$$\mathrm{CF}=\frac{\mathrm{Msample}}{\mathrm{Mbackground}}$$

where Msample and Mbackground are heavy metal concentrations of the studied soil sample and the background, respectively, the Mbackground was taken for As, Cd, Cr, Cu, Pb, Zn, Ni, and Hg were 13.4, 1, 69, 39, 17, 67, 55, and 0.08 mg/kg, respectively (Taylor and McLennan 1995). Hakanson (1980) classified contamination levels as CF 1 (low contamination), CF 3 (moderate contamination), CF 6 (substantial contamination), and CF > 6 (severe contamination) (very high contamination).

#### Degree of contamination (CD)

To make pollution management easier, Hakanson (1980) introduced the “degree of contamination” (CD) as an investigative tool and proposed a classification for contamination degree as CD 6 (low degree of contamination), CD 6 12 (moderate degree of contamination), CD 12 24 (considerable degree of contamination), and CD > 24 (high degree of contamination), indicating alarming anthropogenic pollution. It was specified as the total of the CF of each element involved, as follows:

$$\mathrm{Cdeg}=\sum\limits_{i=1}^{n}\mathrm{CFi}$$

where CFi is the contamination factor for each studied element (i). This index categorizes soil pollution into four categories: low degree of contamination (Cdeg ≤ 8), moderate degree of contamination (8 ≤ Cdeg ≤ 16), significant degree of contamination (16 ≤ Cdeg ≤ 32), and very high degree of contamination (32 ≤ Cdeg) Hakanson (1980); Caeiro et al. (2005).

#### Modified degree of contamination (mCd)

According to Abrahim and Parker (2008), Machenderet al. (2011), and Rahman et al. (2012), the modified degree of contamination (mCd) is the average of all trace element pollution indices (Cfi), assuming at least three chemical elements (*n*) are utilized in the computations.

Klik et al. (2020) classify soil contamination on a seven-order scale: very low contamination (mCdeg ≤ 1.5), low contamination (1.5 ≤ mCdeg ≤ 2), moderate contamination (2 ≤ mCdeg ≤ 4), high contamination (4 ≤ mCdeg ≤ 8), very high contamination (8 ≤ mCdeg ≤ 16), extremely high contamination (16 ≤ mCdeg ≤ 32), and ultra-high contamination (32 ≤ mCdeg). The following equation is used to compute this index:

$$\mathrm{mCdeg}=\frac{1}{n}\sum\limits_{i=1}^{n}\mathrm{CFi}$$

where *n* is the number of elements evaluated and CF is the contamination factor for each element (*i*) individually.

#### Geo-accumulation index (Igeo)

The geo-accumulation index (Igeo) has been widely used as a geochemical criterion to assess the contamination level of a particular element in environmental sediments or soils since 1969. The following equation is used to determine Igeo values. Muller, (1969): $\mathrm{Igeo}={\mathrm{log}}_{2}(\frac{\mathrm{Cn}}{1.5*\mathrm{Bn}})$

Cn is the measured element concentration in the soil, and Bn is the provided metal’s geochemical background value. The constant 1.5 is a background matrix adjustment factor considering natural oscillations caused by lithogenic influences. The background values used in this investigation for As, Cd, Cr, Cu, Pb, Zn, Ni, and Hg were 13.4, 1, 69, 39, 17, 67, 55, and 0.08 mg/kg, respectively (Taylor and McLennan 1995).

The Igeo is divided into seven levels: Igeo ≤ 0, uncontaminated; 0 < Igeo ≤ 1, uncontaminated to moderately contaminated; 1 < Igeo ≤ 2, moderately contaminated; 2 < Igeo ≤ 3, moderately to heavily contaminated; 3 < Igeo ≤ 4, heavily contaminated; 4 < Igeo ≤ 5, heavily contaminated to extremely contaminated; and 5 ≤ Igeo, extremely contaminated (Chen et al. 2015; Hussain et al. 2015; Wei and Yang 2010).

#### Pollution load index (PLI)

A pollutant load index is a measure used to assess the quality of soil (Liu et al. 2005; Santos et al. 2020; Karki and Verma 2020; Tomlinson et al. 1980) determined as follows: by taking the *n*th root of the multiplication of the contamination factors of the examined chemical elements.

$$PLI =({CF}_{1}\times {CF}_{2}\cdots \cdots {{CF}_{n})}^{1/n}$$

where CF_1_, CF_2_, and CF_*n*_ are the contamination factors for elements 1, 2, and *n*, respectively. This indicator divides the soil into three categories: contaminated (PLI > 1), baseline contamination (PLI = 1), and unpolluted (PLI < 1).

#### Pollution index

The pollution index (PI) is defined as the ratio of heavy metal concentration to the geometric mean of background concentrations, and it is used to determine the amount of pollution of particular elements (Keshav Krishna and Rama Mohan 2016; Chenet al. 2005; Sun et al. 2010). The calculation of PI is as follows:

$$\mathrm{PI}=\sqrt{\frac{\left(\mathrm{CFaverage}\right)2+(\mathrm{CFmax})2}{2}}$$

where CFmax is the maximum value of the contamination factors and CFaverage is the average value of the contamination factor. The PI value of each metal of each sample site is calculated and classified respectively as low contamination (PI ≤ 1.0), moderate contamination (1.0 < PI ≤ 3.0), or high contamination (PI > 3.0) (Keshav Krishna and Rama Mohan 2016; Wei and Yang 2010; Chen et al. 2005).

#### Biochemical analysis of enzymes

According to the significance of biochemical biomarkers in the detection of organisms’ exposure to chemicals and environmental pollution, which are typically more sensitive and represent early observable reactions to environmental changes (McCarthy and Shugart 1990; Benson and DiGiulio 1992; Huggett et al. 1992; Mayer et al. 1992; Lagadic et al. 1994; Domingues et al. 2010; Praet et al. 2014), the present research suggested using particular enzymes such as acetylcholinesterase enzyme (AchE) and glutathione-s-transferase (GST) for pollution monitoring as suitable biomarkers in sample preparation and analysis.

#### Sample preparation and analysis

Whole body insects were weighed and homogenized in a saline solution (1 gm of tissue insect/1 mL saline solution 0.7%) using a fine polytron homogenizer for 2 min. The homogenates were then ice-centrifuged at 4000 r.p.m for 15 min. The supernatant was used directly or frozen until the use for the measurement of each enzyme activity. Three replicates were used for these measurements at each one.

#### Acetylcholinesterase

According to the technique adopted by Knedel and Böttger (1967), samples were assayed using a commercial kit to measure the activity of AchE concentrations by the company of Bio Diagnostic Company, Egypt.

#### Glutathione-S-transferases

Glutathione-S-transferases activity (GST) was measured according to Habig et al. (1974) using 1-chloro-2, 4 dinitrobenzene (CDNB) as a substrate with reduced glutathione. The conjugation is accompanied by an increase in absorbance at 340 nm. The rate of increase is directly proportional to the GST activity in the sample.

### Statistical analysis

The statistical software SPSS V.22 was used to code and input the data. Data were tested for satisfying assumptions of parametric tests; continuous variables were subjected to Shapiro–Wilk and Kolmogorov–Smirnov tests for normality. Probability and percentile data were standardized for normality using Arcsine Square Root. Data were presented as mean and standard deviation. ANOVA analyses were done for the investigated sites regarding the recorded variables; analysis was evaluated using three replicates at least for each group; post hoc analysis was assessed using Tukey pairwise comparison using MiniTab V 14; *P* values were considered significant at < 0.05. Regression was conducted to figure out the relation and the prediction equation between soil heavy metals and *C. savignyi* detoxification enzyme; the analysis became available using SigmaPlot V 14.0. Distance cluster and CCA were illustrated using Past V 4.12. Data were visualized when possible, using R studio V 2022.02.4.

## Results

### Climatic factors

Regarding the recorded climatic factors in the meteorological station (Table 1), there was a significant seasonal climatic fluctuation (*P* < 0.05) in observed climatic factors. The highest recorded temperature was in summer, 29.4 ± 0.3 °C. On the contrary, winter is characterized by its lower temperature (13.82 ± 0.8 °C). On the other hand, the maximum value of relative humidity was recorded during summer (72.73 ± 0.45%), and minimum relative humidity (68.7 ± 1.15%) was recorded during winter. Furthermore, the highest recorded wind speed was during spring (5.33 ± 0.48 m s^−1^), and the lowest (3.45 ± 0.36 m s^−1^) in winter. Meanwhile, the maximum reported pressure was recorded during winter (101.90 ± 0.5 kPa) and the lowest (100.61 ± 0.4 kPa) in summer.

**Table 1:** The microclimatic factors throughout the study period

Season	Temp (°C)	R.H (%)	W. speed (m s^−1)^	Pressure (kPa)
Summer	27.28 ± 0.11^a^	72.73 ± 0.45^a^	5.29 ± 0.51^a^	100.61 ± 0.4^b^
Autumn	20.2 ± 0.15^c^	70.02 ± 1.46^a, b^	3.74 ± 0.27^b^	101.65 ± 0.21^a^
Winter	16.47 ± 0.18^d^	68.7 ± 1.15^b^	3.45 ± 0.36^b^	101.90 ± 0.5^a^
Spring	22 ± 0.31^b^	68.81 ± 1.69^b^	5.33 ± 0.48^a^	101.59 ± 0.05^a^

Means in a column that shares the same letters is not significantly different (*P* > 0.05)

### Floral coverage

**Fig. 2:**
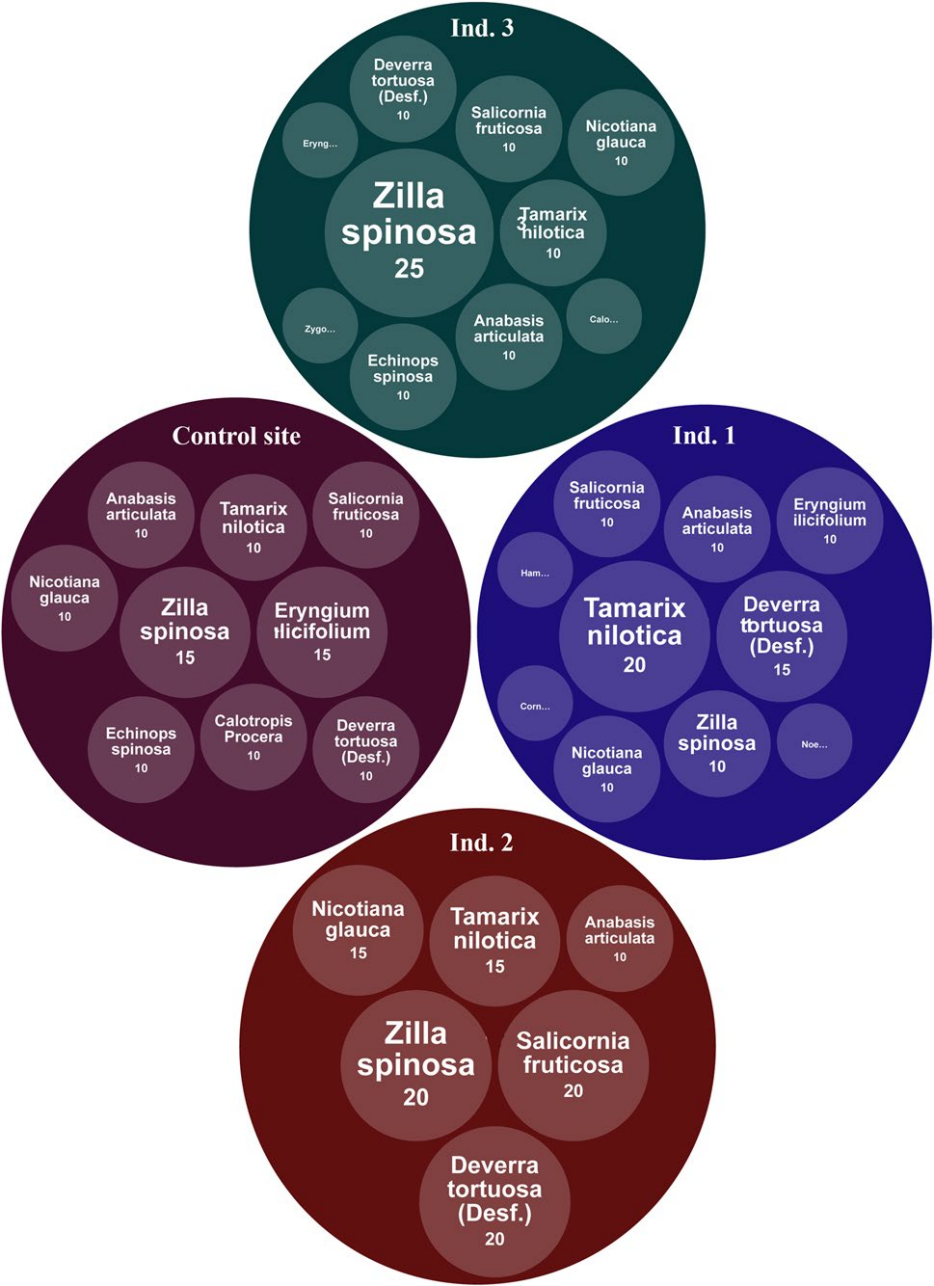
The circle chart represents recorded floral coverage for study sites, ordered from the highest to the lowest

Vegetation coverage is characterized by diversity and species abundance fluctuation. Fourteen plant species were identified (Table 1 (Supplementary file) and Fig. 2) at different study sites in Borg El-Arab. *Zilla spinosa* (Family: Brassicaceae), *Tamarix nilotica* (Family: Tamaricaceae), *Anabasis articulate*, *Salicornia fruticose* (Family: Amaranthaceae), *Deverra tortuosa* (Family: Apiaceae), and *Nicotiana glauca* (Family: Solanaceae) are the most recorded species in study sites. In particular, *Zilla spina* was the dominant species in all vegetation cover at various study sites. Its relative vegetation cover ranged between 10 and 25%. On the other hand, plant community is characterized by a high variety of plant species (14 varied species), with each of their relative vegetation covering ranging between 5 and 25%.

### Soil properties

**Fig. 3:**
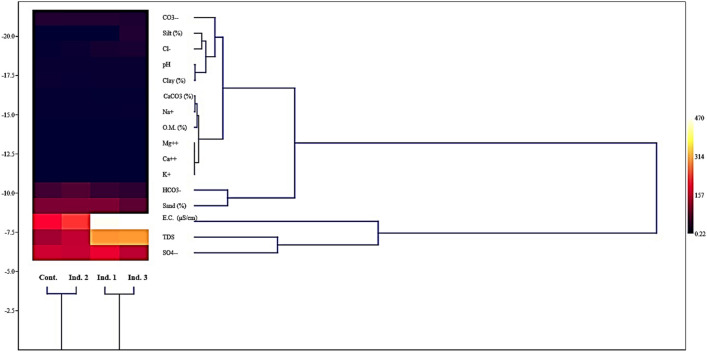
Distance cluster for studied sites regarding their soil characteristics

Table 2 shows spatial variation of physical and chemical properties of soil samples at different study sites. The sandy soil was the main soil type at different study sites except industrial 3 at Borg El-Arab, characterized by its sand, clay, and loam mixture. The percentage of sand, silt, and clay was detected according to the size of soil particles. For soil texture, the sand (%) varied from 93.11 ± 0.15 at industrial 2 to 68.32 ± 0.94 at industrial 3. The soil’s pH was alkaline at all study sites except industrial 3. On the other hand, the results indicated that the electrical conductivity of (EC) recorded its maximum value at industrial 3 (470.31 ± 6.2). Soil texture (sand, clay, and silt %) at all industrial sites varied significantly (*P* < 0.05) with the control site. For PH, TDS, EC, CL^−^, HCO_3_^−^, SO_4_^−−^, and CaCo_3_, all industrial sites were significant (*P* < 0.05) compared to the control site. Na^+^, Ca^++^, Mg^++^, and O.M were significant (*P* < 0.05) for industrials 1 and 3 in comparison to the control site; meanwhile, industrial 2 was insignificant (*P* > 0.05) with the control site. For CO_3_^−−^ industrials 2 and 3 were significant (*P* < 0.05) with the control site. Finally, K at industrial site 3 was significant (*P* < 0.05) with the control site. Figure 3 revealed more similarity between industrials 1 and 3 and dis-similarity compared to industrial 2 and the control site regarding their soil characteristics.

**Table 2:** Spatial variations in the physical and chemical characteristics of the soil samples collected from different industrial sites throughout the current study in Borg El-Arab, Egypt

Site	Type class	Soil texture			pH	TDS	E.C. (μS/cm)	Ion concentration (%)				Extractable cations (mg/100 g dry soil)				CaCO_3_ (%)	O.M. (%)
		Sand (%)	Silt (%)	Clay (%)				Cl^−^	HCO_3_^−^	CO_3_^−−^	SO_4_^−−^	Na^+^	K^+^	Ca^++^	Mg^++^		
Cont	Sand	91.8 ± 0.07^a^	0.58 ± 0.03^a^	7.6 ± 0.07^a^	7.55 ± 0.04^a^	120 ± 2^a^	187.5 ± 2.3^a^	3.5 ± 0.04^a^	48.5 ± 2.7^a^	25.5 ± 0.5^a^	152.2 ± 2.5^a^	3.4 ± 0.16^a^	0.23 ± 0.017^a^	0.37 ± 0.013^a^	0.33 ± 0.013^a^	3.35 ± 0.11^a^	1.03 ± 0.02^a^
Ind. 1	Sand	92.14 ± 0.09^b^	0.76 ± 0.05^b^	7.09 ± 0.06^b^	7.3 ± 0.07^b^	298 ± 4^a^	465.62 ± 4.9^a^	15.75 ± 0.13^b^	40.5 ± 1.8^b^	25 ± 0.6^a, b^	173.8 ± 4.2^b^	3.2 ± 0.13^b^	0.22 ± 0.011^b^	0.41 ± 0.018^b^	0.38 ± 0.021^a^	2.75 ± 0.07^b^	0.51 ± 0.015^a^
Ind. 2	Sand	93.11 ± 0.15^c^	1.02 ± 0.07^c^	5.85 ± 0.04^c^	7.5 ± 0.06^c^	144 ± 2^b^	225 ± 3.4^b^	8.75 ± 0.07^c^	60 ± 2.7^c^	24.5 ± 0.45^b^	146.2 ± 2.25^c^	3.6 ± 0.2^b, c^	0.22 ± 0.014^b^	0.36 ± 0.019^b^	0.32 ± 0.024^b^	3.9 ± 0.15^c^	1.03 ± 0.011^b^
Ind. 3	Sandy clay loam	68.32 ± 0.94^d^	24.23 ± 0.3^d^	7.44 ± 0.02^d^	6.9 ± 0.05^d^	301 ± 5^c^	470.31 ± 6.2^c^	18.55 ± 0.45^d^	34 ± 1.3^d^	21.5 ± 0.35^c^	139 ± 3.2^d^	4.7 ± 0.43^c^	0.33 ± 0.016^b^	0.27 ± 0.023^c^	0.38 ± 0.019^b^	3.3 ± 0.13^d^	0.68 ± 0.008^c^

Means in a column that shares the same letters is not significantly different (*P* > 0.05)

**Fig. 4:**
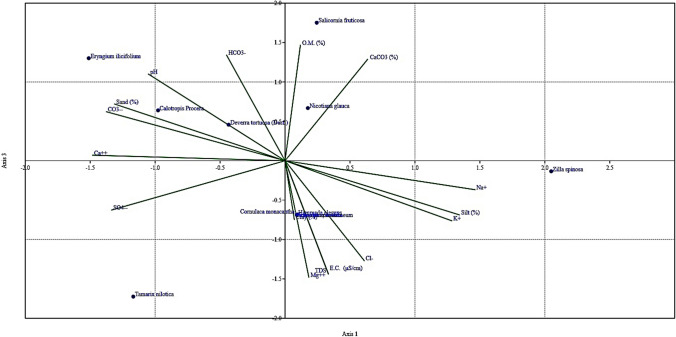
CCA represents soil properties’ role in floral distribution and coverage

Floral coverage depends directly on soil properties that simultaneously provide suitable habitats for vegetation. On the other hand, the plant distribution reflects those properties as they are associated with each site’s soil characteristics (Fig. 4).

### Heavy metal in soil

Heavy metals in soil were measured (Table 3 and Fig. 1 (Supplementary file)) as arsenic (As), lead (Pb), nickel (Ni), and mercury (Hg) at investigated sites, and Hg concentration varied significantly (*P* < 0.05) for industrials 2 and 3 in comparison to the control site. Pb and Ni concentrations statistically varied (*P* < 0.05) between industrials 1 and 3 to the control site.

**Table 3:** Spatial variations in the values of heavy metals (ppm) of the soil samples collected from different industrial sites throughout the current study in Borg El-Arab, Egypt

Site	As (ppm)	Pb (ppm)	Ni (ppm)	Hg (ppm)
Cont	40 ± 2.82^b^	1.35 ± 0.21^c^	29.5 ± 2.12^c^	1 ± 0.14^b^
Ind. 1	33.5 ± 3.53^b^	2.25 ± 0.07^b^	37 ± 2.41^b^	0.85 ± 0.07^b^
Ind. 2	18.5 ± 0.7^c^	1.15 ± 0.06^c^	32.5 ± 0.7^c^	0.55 ± 0.04^c^
Ind. 3	92 ± 4.24^a^	6.5 ± 0.14^a^	95 ± 1.41^a^	3.25 ± 0.27^a^

Means in column that share the same letters is not significantly different (*P* > 0.05)

### Pollution indices

#### Contamination factor (Cf)

For As, industrial 3 exhibits a very high contamination factor; meanwhile, industrials 1 and 2 and control sites were a moderate contamination factor. Pb at all sites shows a low contamination factor. Ni at all sites exhibits a low contamination factor except industrial 3, which is moderate. Finally, Hg at all sites exhibits a high contamination factor (Fig. 6a).

#### Ecological risk factor (E_r_)

The risk factor was successfully used for assessing the contamination of soils in the environment by heavy metals. Regarding As, industrial 3 was at considerable ecological risk; meanwhile, industrials 1 and 2 were at low ecological risk, while control exhibited a moderate ecological risk. Pb and Hg at all sites represent a low ecological risk. Regarding Ni, all sites were at low ecological risk except at industrial 3, which exhibits a considerable ecological risk (Fig. 6a).

#### Geo-accumulation index (Igeo)

For As, industrial 3 was moderately polluted; meanwhile, industrials 1 and 2 and control sites were unpolluted to moderately polluted. For Pb, all sites were unpolluted. Regarding Ni, all sites were unpolluted to moderately polluted. Finally, Hg at all sites was moderately polluted, except industrial 3 which was moderately to heavily polluted (Fig. 6a).

#### Degree of contamination (Cdeg)

The degree of contamination at industrial 3 was at a very high degree of contamination, while industrial 1 and 2 had a moderate degree of contamination, while the control site had a considerable degree (Fig. 6b).

#### Modified degree of contamination (mCd)

**Fig. 5:**
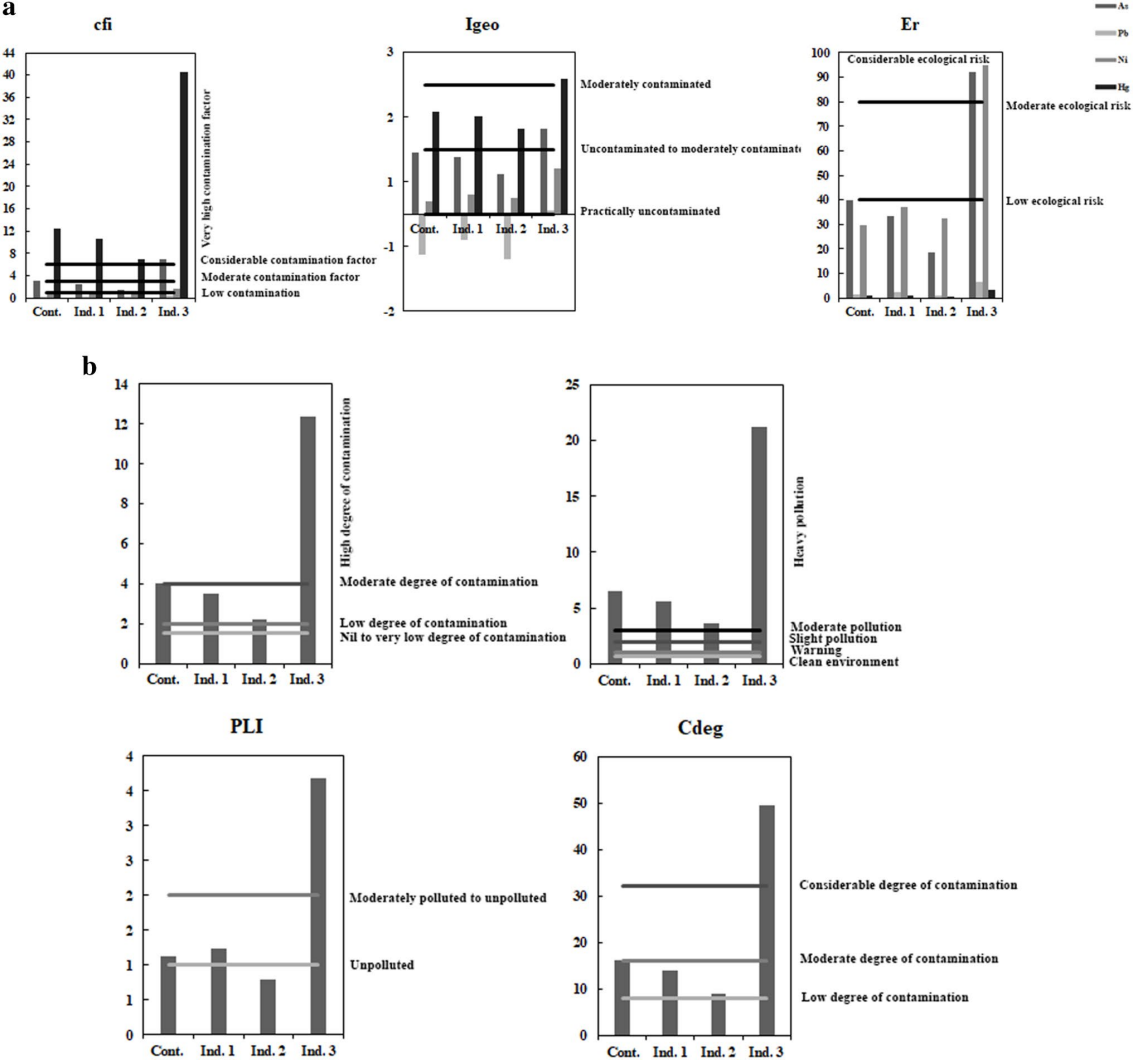
Pollution indices calculated for soil’s heavy metal at studied sites. **a** Cf, Igeo, and Er. **b** mcd, Pi, PLI, and Cdeg

Industrial 3 represents a very high degree of contamination, while industrials 1 and 2 and control sites show moderate contamination (Fig. 5b).

#### Pollution load index (PLI)

**Fig. 6:**
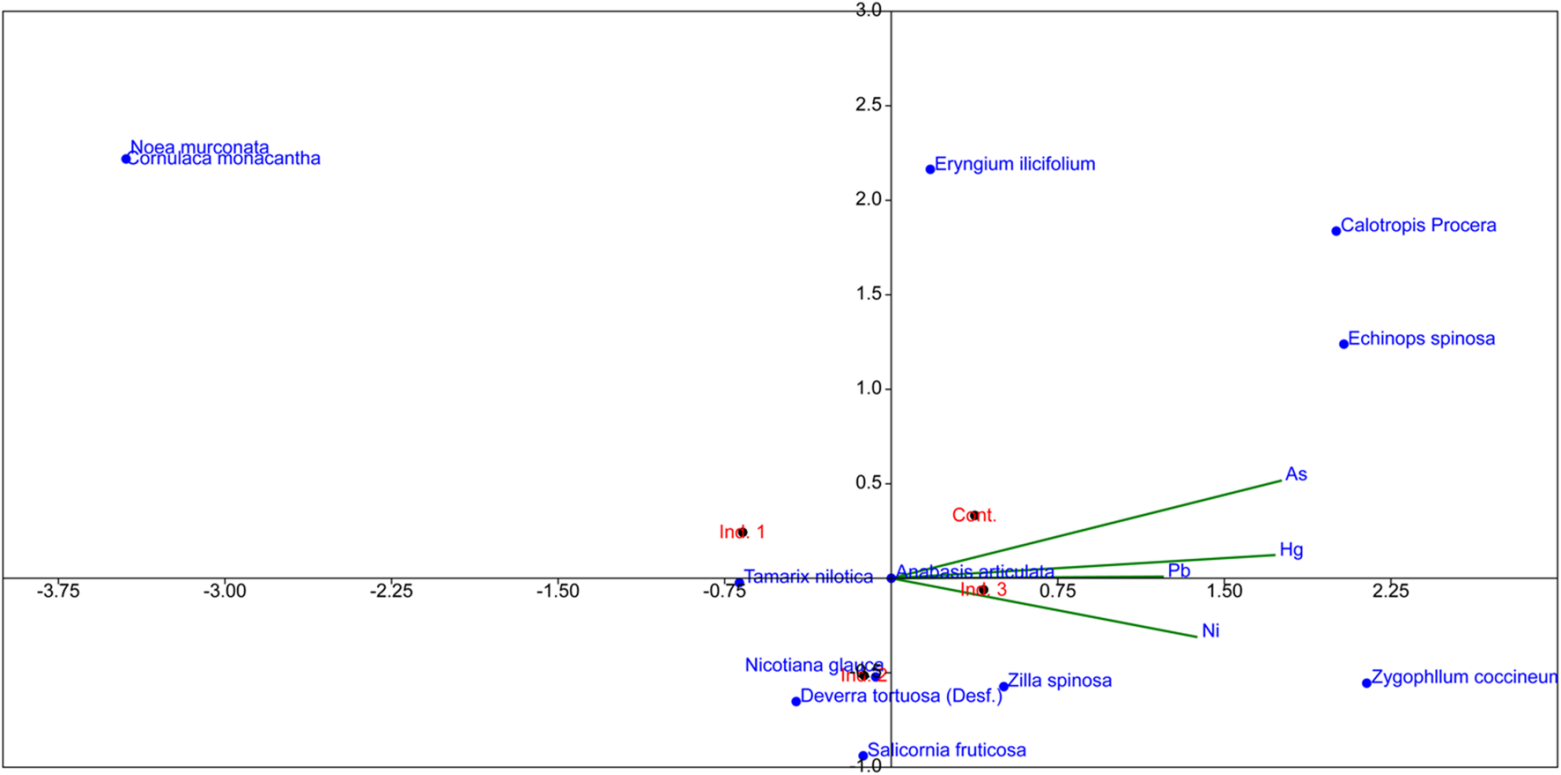
CCA correlation between soil heavy metals relation to floral coverage at different study sites

According to PLI, industrial 3 was moderately to highly polluted, while industrial 2 was unpolluted, but industrial 1 and the control sites were moderately polluted to unpolluted (Fig. 6b).

#### Pollution index (PI)

According to PI indices, all studied sites were heavily polluted (Fig. 5b).

### Heavy metal contamination on floral coverage

Soil heavy metals show a direct relation to floral coverage, as shown in Fig. 6; the effect of heavy metals followed the following order As > Hg > Ni > Pb. This reflects the present observation of floral coverage, with As the highest effective heavy metal alters and the floral coverage’s role in polluted stations.

### Enzymatic activity

**Fig. 7:**
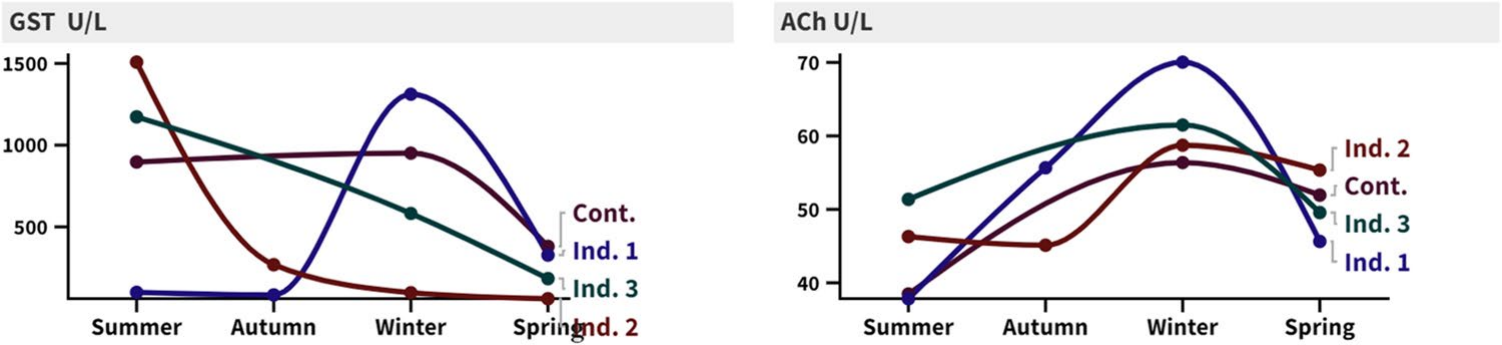
GST and ACh enzymatic activities recorded for *C. savignyi* at investigated sites during the period of the study

Enzymatic activity of (GST and ACh) for *C. savignyi* have been observed as a biomarker for soil contamination at investigated sites, which reveals a significant seasonal variation (*P* < 0.05) over the period of the study for both enzymes; this change could be a direct effect of soil recorded heavy metals (Fig. 7). The observed soil contamination rolled the enzymatic activity of (GST and ACh) of *C. savignyi* as following equations:

$$\mathrm{GST }(\mathrm{U}/\mathrm{L})=-484.2 + 16.76\mathrm{ As }- 853.1\mathrm{ Pb }+ 54.38\mathrm{ Ni}$$

$$\mathrm{ACh }(\mathrm{U}/\mathrm{L})= 51.94- 0.1473\mathrm{ As }+ 3.013\mathrm{ Pb }- 0.04039\mathrm{ Ni}$$

## Discussion

Developing practical techniques for avoiding or limiting potentially disastrous implications for biodiversity became vital. An agricultural environmental program is an additional instrument that has the potential to offset the negative trends of losing biodiversity (Pereira et al. 2012). Environmental monitoring is required for ecosystem management since industrial pollution directly damages ecosystems. The phrase “bioindication” does not have a conventional definition; rather, it alludes to a growing issue in the assessment of conservation efforts. A “bioindicator” is a species or collection of species representing the environment’s abiotic or biotic status. It displays how environmental change affects a habitat, community, or ecosystem and determines whether the change is good or harmful (Parmar et al. 2016). Environmental changes that impair essential activities such as metabolism, development, and reproduction are particularly sensitive to many living creatures (David 1989).

Fourteen plant species were identified at 4 study sites in the Borg El-Arab district (one control site and three industrial sites). *Zilla spinosa* (Family: Brassicaceae), *Tamarix nilotica* (Family: Tamaricaceae), *Anabasis articulate*, *Salicornia fruticose* (Family: Amaranthaceae), *Deverra tortuosa* (Family: Apiaceae), and *Nicotiana glauca* (Family: Solanaceae) are the most recorded species in study sites. In particular, *Zilla spina* was the dominant species in all vegetation cover at different study sites, which agrees with Bream et al. (2019). Due to their large biomass, rapid growth, and capacity to adapt to extreme climates in nature, native vegetation on mining sites should be used as much as possible as a better indicator of reclamation effectiveness (Bandyopadhyay 2022). Heavy metals in soils function as micronutrients, yet at larger concentrations, they hinder or slow plant development and its planned metabolic activities (Dankoub et al. 2012; Taghipour et al. 2011). Depending on the plant’s genetic imprint and genotype, certain plants may handle high levels of heavy metals (Leitenmaier and Küpper 2013; Pulford and Watson 2003; Jamal et al. 2006).

The physicochemical characteristics of the soil samples for the analyzed locations demonstrated that the electrical conductivity in industrials 3, 1, and 2 average (470.31 ± 6.2, 465.62 ± 4.9, and 225 ± 3.4 μS/cm, respectively) was significantly higher than that in the control site (187.5 ± 2.3 μS/cm). Soil electrical conductivity (EC) is essential to soil health; it measures soil management and environmental media for long-term sustainability. The findings are congruent with Adviento-Borbe et al. (2006), who stated that soil EC influences soil microbial activity, plant nutrient availability, and agricultural yields. Cropping, irrigation, land usage, and the use of fertilizer, manure, and compost all have an impact on it.

Soil pH values of industrial 3 (6.9 ± 0.05) were significantly lower than the control site (7.55 ± 0.04). This finding is related to a rise in As, Pb, and Hg levels, which decreases soil pH and reflects heavy metal desorption from the soil. Soni (2016) achieved similar results. According to Arias et al. (2005), soil pH (alkalinity and acidity) indicates the most effective parameter for nutrient availability to plants, insects, and other organisms in the soil. In addition, Kekane et al. (2015) found that pH levels affect metal solubility and plant availability. Our findings revealed that the chloride concentration of the industrial 3 soil sample (18.55 ± 0.45%) was significantly higher compared to the control site (3.5 ± 0.04%). According to Schulte (1999), soil acidity, organic matter, and aeration all influence chloride accessibility to plants. Because chlorine is a vital component of photosynthesis, high limits of chloride ions cause higher concentrations in plant take-up, producing crop toxicity concerns and a resultant drop in production.

Toxic metals may accumulate in the soil due to industrial emissions, fertilizer and pesticide application, petrochemical leakage, wastewater irrigation, and atmospheric deposition (Khan et al. 2008; Zhang et al. 2010). As levels of heavy metal contamination in soil grow, they may alter, transfer to plants, and be transmitted from plants to animals and humans (Atayese et al. 2010). As a result, heavy metal buildup in soil was identified at the present research locations. The present investigation of heavy metals in soil discovered the following accumulation order from high to low concentrations: Ni > As > Pb > Hg. Industrial 3 had a higher heavy metal level than the control site. This conclusion is attributable to the growing emission of heavy metals in industries more than others, which fits with Bream et al. (2019), who indicated that metal increases are connected with industrial pollution sources. Ni levels in surface soils indicate soil formation and contamination. Alloys, chemical industries, petroleum refining, batteries, waste disposal, sewage sludge, fertilizer, transportation, fuel, and coal combustion are the primary anthropogenic sources of Ni (Reimann and De Caritat 1998). Anthropogenic activities are the primary source of soil arsenic (Bohn 1979). Arsenic is abundantly spread in nature. As concentrations in the soil samples tested ranged from 18.5 to 92 ppm. This is more than the global soil content (5 ppm) (Kabata-Pendias and Mukherjee 2007). The lead concentration of soil has been found to vary from 1.15 to 6.5 ppm. Pb concentrations in our environment have significantly grown and are gradually accumulating in the surface soil strata where man has utilized it for over 5000 years (Bradl et al. 2005). It is in batteries, pigments, plastic stabilizers, ammunition, special alloys, pipes, and solder; its organic compounds are in insecticides and as an antiknock agent in leaded gasoline (Reimann and De Caritat 1998). On the other hand, anthropogenic sources of Hg include cement manufacture, the chemical and pharmaceutical industries, coal combustion, and municipal solid waste incineration. Other possible indoor sources include building materials (interior decorations, paints, and fluorescent lamps), home appliances and electrical gadgets, LCDs, monitors, batteries, clothes dryers, irons, washing machines, fluorescent bulbs, neon lights, and thermometers (Behrooz et al. 2022).

The results of contamination factor (CF): average CF values for heavy metals have an order Hg > As > Ni > Pb. Hg at all sites exhibits a high contamination factor. For As, industrial 3 exhibits a very high contamination factor; meanwhile, industrials 1 and 2 and control sites were a moderate contamination factor. Ni at all sites exhibits a low contamination factor except industrial 3, which is moderate. Finally, Pb at all sites shows a low contamination factor. Cdeg estimated values range from 8.9 to 49.6 in soil samples, with Cdeg at industrial 3 indicating a significant level of contamination. In comparison, industrials 1 and 2 had moderate pollution, but the control site had significant contamination. Igeo was computed for all metals using Taylor and McLennan’s (1995) background values since no background values for these items are declared in this city. The geo-accumulation index (Igeo) for the heavy metals investigated was arranged as follows: Hg > As > Ni > Pb in the soil samples. The risk factor was successfully used for assessing the contamination of soils in the environment by heavy metals. The Er values for the nickel varied from 29.5 to 95, evidencing a moderate ecological risk from this metal for all sites except industrial 3 considerable ecological risks. Arsenic varied from 18.5 to 92, evidencing a moderate ecological risk from this metal for all sites except industrial 3 considerable ecological risks. The lead and mercury results are lower than 40, representing a low ecological risk. Industrial 3 represents a very high degree of contamination, while industrials 1 and 2 and control sites show moderate contamination. The PLI values ranged from 0.78 to 3.68, indicating that industrial 3 was moderate to highly polluted, while industrial 2 was unpolluted, but industrial 1 and control sites were moderately polluted to unpolluted. PI is a quick tool and guide for comparing pollution status from a unique soil environment (Mazurek et al. 2017). According to PI indices, all studied sites were heavily polluted. The difference in indices results is due to the difference in sensitivity of these indices towards the soil pollutants (Praveena et al. 2007).

Detoxification enzymes in insects are an enzymatic defense against foreign chemicals and are important in maintaining normal physiological activities (Xiaozhen and Yinghong 2010). Many aquatic creatures have defensive mechanisms to resist reactive oxygen species (ROS), such as glutathione-s-transferase (GST) and acetylcholinesterase (AChE), which have detoxifying capabilities against lipid hydroperoxides formed by organic contaminants as well as heavy metals (Farombi et al. 2007). GST enzyme is also considered an antioxidant, facilitating their conjugation with GSH and producing non-toxic compounds (Xu et al. 2015). The present findings eliminated an inhibition of GST levels in *C. savignyi* at the industrial site compared to the control site. This outcome is consistent with Sun et al. (2016); they established that GST activity reduced with an increase in the HM in the beetle’s body burdens while increasing with the addition of xenobiotics. Also, Migula et al. (2004) proved that correlations between GST activity and body loads of HMs were positive with Cu in *G. stercorosus* and *S. caesareus* and negative with Zn in *P. oblongopunctatus* and Cd in *S. caesareus*. According to Terriere (1984), GST constitutes 85% protein; hence, GST decrease in greater polluted areas corresponds to an increase in the protein level of the insect. Furthermore, GST activity linked positively with Pb, Mn, and Cu while negatively with Ni and Zn. It might be attributed to HMs in Egyptian beetle samples living in polluted locations, preventing active centers of an enzyme. The current findings contradicted Stone et al. (2002), who found no significant variation in GST activity in male ground beetles collected from metal-polluted locations.

However, AChE activity was shown to be greater in industrial settings than in controls, possibly due to heavy metals increasing acetylcholine at synapses, causing the postsynaptic membrane to be permanently stimulated. Acetylcholinesterase (AChE) is a protein that is responsible for the breakdown of acetylcholine into choline and acetic acid in cholinergic synapses and neuromuscular junctions in both vertebrates and invertebrates (Pena-Lopis et al. 2003). This was consistent with the findings of Gill et al. (1991), who discovered enhanced AChE activity in the skeletal muscles and brain of a fish species exposed to Cd for 48 h. Additionally, Bream et al. (2019) discovered increased AChE activity in heavy and medium industrial sites in El-Sadat, Egypt. Zatta et al. (2002) also discovered enhanced AChE activity in rats given alumina orally. In contrast, Lavado et al. (2006) found that organophosphorus, carbamates, and heavy metals substantially inhibited AChE in the muscle of several invertebrates collected from a polluted river. Furthermore, activity suppression was linked to Zn, Cu, and Cd buildup in treated clams’ digestive glands and gills (Kamel et al. 2012).

## Conclusion

The study demonstrated that insects could serve as effective bioindicators of heavy metal pollution and environmental changes resulting from industrial impacts. As sensitive organisms, they may provide early warning signals of ecosystem alterations. Vegetation surveys revealed *Zilla spina* as the dominant plant species across sites. Electrical conductivity and heavy metal content in soils, particularly nickel, arsenic, lead, and mercury, were significantly elevated at industrial locations compared to the control site. Calculated contamination degree and pollution load index scores uncovered a profound level of contamination correlating with chemical and silicate factory activity for one industrial site. Conversely, the control demonstrated a moderate contamination score despite being distanced from human influences. Biomarker analyses highlighted suppressed glutathione S-transferase levels in *C. savignyi* at the industrial site relative to controls, inferring toxicity. However, acetylcholinesterase activity was paradoxically increased, potentially reflecting heavy metal stimulation of synaptic acetylcholine signaling. Collectively, these enzymatic biomarkers permit effective environmental monitoring and assessment of anthropogenic contamination.

### Supplementary Information

Below is the link to the electronic supplementary material.

Supplementary file1 (DOCX 194 KB)

## Data Availability

The datasets used and/or analyzed during the current study are available from the corresponding author on reasonable request.
