# Supplementary material for: Environmental impacts of industrial activities on floral coverage with special emphasis on detoxification enzyme activities in Cataglyphis savignyi as pollution biomarker
Source: Environ Sci Pollut Res Int. 2023 Oct 18;30(53):113758–73. doi: 10.1007/s11356-023-30367-1 (PMC10663209; doi:10.1007/s11356-023-30367-1)
Supplement: Supplementary file 1 — Supplementary file1 (DOCX 194 KB) [file 11356_2023_30367_MOESM1_ESM.docx]

**Table (1. Supplementary file):** Spatial variations of identified plant species (presence; ✔) and their relative vegetation cover (value %) at different industrial sites throughout the study period.

| Scientific name | Family | Borg El-Arab | | | |
| --- | --- | --- | --- | --- | --- |
|  |  | **Cont.** | **Ind. 1** | **Ind. 2** | **Ind. 3** |
| *Zilla spinosa* | Brassicaceae | **✔ (15%)** | **✔ (10%)** | **✔ (20%)** | **✔ (25%)** |
| *Calotropis Procera* | Apocynaceae | **✔ (10%)** |  |  | **✔ (5%)** |
| *Tamarixnilotica* | Tamaricaceae | **✔ (10%)** | **✔ (20%)** | **✔ (15%)** | **✔ (10%)** |
| *Anabasis articulata* | Amaranthaceae | **✔ (10%)** | **✔ (10%)** | **✔ (10%)** | **✔ (10%)** |
| *Salicornia fruticosa* | Amaranthaceae | **✔ (10%)** | **✔ (10%)** | **✔ (20%)** | **✔ (10%)** |
| *Deverratortuosa* | Apiaceae | **✔ (10%)** | **✔ (15%)** | **✔ (20%)** | **✔ (10%)** |
| *Echinops spinosa* | Compositae | **✔ (10%)** |  |  | **✔ (10%)** |
| *Nicotiana glauca* | Solanaceae | **✔ (10%)** | **✔ (10%)** | **✔ (15%)** | **✔ (10%)** |
| *Noeamurconata* | Chenopodiaceae | | **✔ (5%)** |  |  |
| *Zygophllum coccineum* | Zygophyllaceae | |  |  | **✔ (5%)** |
| *Eryngium ilicifolium* | Apiaceae | **✔ (15%)** | **✔ (10%)** |  | **✔ (5%)** |
| *Cornulacamonacantha* | Chenopodiaceae | | **✔ (5%)** |  |  |
| *Hammada elegans* | Amaranthaceae | | **✔ (5%)** |  |  |

| 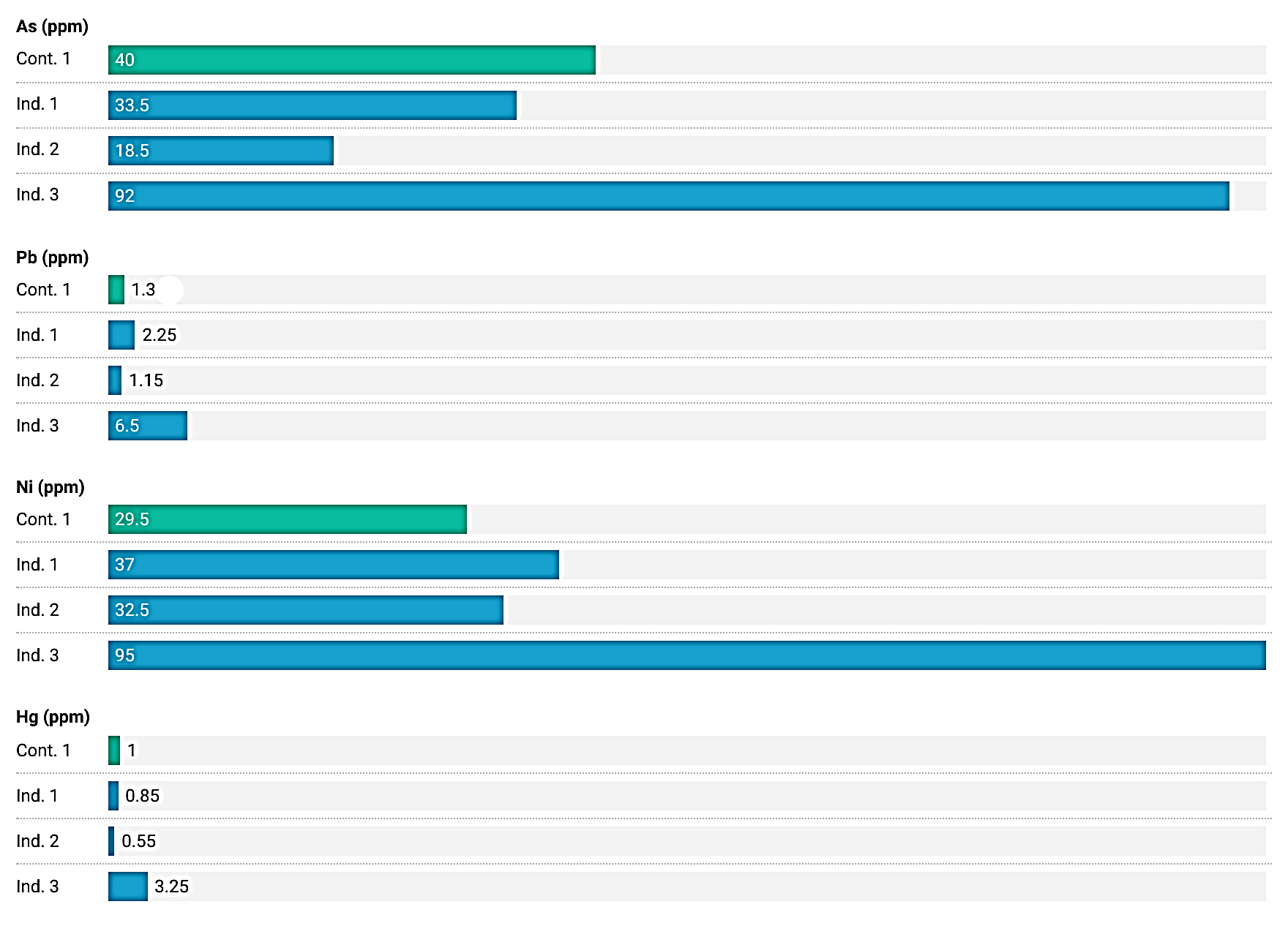 |
| --- |
| Fig (1. Supplementary file): Spatial variations in the values of heavy metals (ppm) of the soil samples collected from different industrial sites throughout the current study. |
